# *Thaumatin-like Protein* (*TLP*) Genes in Garlic (*Allium sativum* L.): Genome-Wide Identification, Characterization, and Expression in Response to *Fusarium proliferatum* Infection

**DOI:** 10.3390/plants11060748

**Published:** 2022-03-11

**Authors:** Olga K. Anisimova, Elena Z. Kochieva, Anna V. Shchennikova, Mikhail A. Filyushin

**Affiliations:** Research Center of Biotechnology, Institute of Bioengineering, Russian Academy of Sciences, Leninsky Ave. 33, Bld. 2, 119071 Moscow, Russia; lelikanis@yandex.ru (O.K.A.); ekochieva@yandex.ru (E.Z.K.); shchennikova@yandex.ru (A.V.S.)

**Keywords:** *Allium sativum* L., thaumatin-like proteins, biotic stress, *Fusarium proliferatum*, gene structure, gene expression

## Abstract

Plant antifungal proteins include the pathogenesis-related (PR)-5 family of fungi- and other stress-responsive thaumatin-like proteins (TLPs). However, the information on the TLPs of garlic (*Allium sativum* L.), which is often infected with soil *Fusarium* fungi, is very limited. In the present study, we identified 32 *TLP* homologs in the *A. sativum* cv. Ershuizao genome, which may function in the defense against *Fusarium* attack. The promoters of *A. sativum* *TLP* (*AsTLP*) genes contained *cis*-acting elements associated with hormone signaling and response to various types of stress, including those caused by fungal pathogens and their elicitors. The expression of *AsTLP* genes in *Fusarium*-resistant and -susceptible garlic cultivars was differently regulated by *F. proliferatum* infection. Thus, in the roots the mRNA levels of *AsTLP7–9* and *21* genes were increased in resistant and decreased in susceptible *A. sativum* cultivars, suggesting the involvement of these genes in the garlic response to *F*. *proliferatum* attack. Our results provide insights into the role of TLPs in garlic and may be useful for breeding programs to increase the resistance of *Allium* crops to *Fusarium* infections.

## 1. Introduction

Plant antifungal proteins provide resistance to fungal pathogens and are a focus of agricultural biotechnology. Overall, 13 classes of antifungal proteins are distinguished based on structural similarity and functional activity [1]. Among them, the pathogenesis-related (PR)-5 family members are homologous to the sweet-tasting protein thaumatin isolated from a Western African herb *Thaumatococcus danielli* Benth [2]. Thaumatin-like proteins (TLPs) are identified in more than 180 plants, including dicots, monocots, gymnosperms, bryophytes, and algae [3], and some of them display strong antifungal activity against *Rhizoctonia solani*, *Alternaria alternata*, *Fusarium graminearum*, *Fusarium solani*, *Verticillium* spp., and *Phytophtora* spp. [4,5,6]. TLPs could also be activated by bacterial pathogens, abiotic stresses (such as wounding, drought, osmotic stress, low temperature, high salinity, and UV radiation), and plant hormones [7,8,9,10,11,12,13,14,15].

Constitutive expression of *TLP* genes enhances plant tolerance to fungal pathogens [16,17,18,19,20,21,22,23,24]. Thus, transgenic tobacco plants overexpressing peanut, rice, or cotton TLPs show increased resistance to *Botrytis cinerea*, *R. solani*, *Fusarium oxysporum*, *F. solani* [23], *A. alternata* [17], and *Verticillium dahliae* [24], whereas transgenic potatoes overexpressing the *Camellia sinensis TLP* gene are resistant to *Phytophthora infestans* [19]. It has been reported that TLPs exert antifungal effects by lysing the fungal cell wall and inhibiting hyphal growth [25,26], which could be due to the β-glucanase activity of TLPs as well as to their role in the induction of pathogen defense-related mechanisms, including phenylpropanoid and phytoalexin production [10,18,24].

Interestingly, TLPs can also be expressed by plant pathogens as a part of their molecular mimicry strategy to evade the plant’s defense system and promote colonization of the host [27]. For example, TLPs of the nematode *Bursaphelenchus xylophilus* (Bx-TH1 and Bx-TH2) transiently expressed in *Nicotiana benthamiana* induce plant cell death [28,29].

TLPs are also known as pollen and fruit allergens, which stimulate IgE production, thus predisposing people to allergy [30,31,32].

As a group, TLPs represent highly soluble low molecular weight proteins classified into L-type (22–26 kDa) and S-type (<18 kDa) [33,34]; according to the isoelectric point (pI), some of them are highly acidic (pI = 3.4), whereas the other—very basic (pI = 12). TLPs are resistant to proteases and heat/pH-induced denaturation because of high stability of their structure due to disulfide bonds formed by 10 (S-type) or 16 (L-type) conserved cysteines [32]. Some plant species express TLPs fused with protein kinases (PR5- or TLP-kinases), which are suggested to serve as receptor protein kinases for pathogen sensing and activation of the downstream signaling [35,36,37,38,39]. Other members of the TLP family are osmotins associated with osmotic regulation, salt stress resistance, and antifungal activity [3,40] and permatins involved in antifungal defense of developing seeds [41,42].

In total, 344 plant TLPs have been identified through analysis of NCBI, EMBL, DDBJ, UniProt, and other databases [43]. Most of them contain an N-terminal signal peptide, specific glycoside hydrolase domain, highly conserved molecular signature motif GX[GF]XCXT[GA]DCX(1,2)GX(2,3)C, REDDD motif, and conserved Cys residues [33]. TLPs have endo-β-1,3-glucanase activity and can hydrolyze β-1,3-glucan of the fungal cell wall by binding to it through a negatively charged acidic interdomain cleft containing conserved residues underlying substrate binding (Lys and two Thr forming hydrogen bonds with (1,3)-β-d-glucan) and cleft acidity (Glu and three Asp) [27,44]. The acidic cleft and a significantly shortened so-called “thaumatin loop” of TLPs are responsible for the disappearance of sweet taste inherent to thaumatin, which has a basic cleft (surrounded with Lys residues replaced in TLPs by neutral Thr, Leu, and Ala and acidic Asp) [27].

The aim of this study was to characterize *TLP* genes in garlic (*Allium sativum* L.), one of the important bulb crops highly susceptible to infection with *F. oxysporum* f. sp. *cepae* (*Fusarium* basal rot, FBR) and *F. proliferatum* (bulb rot), which are responsible for more than 60% of the world’s garlic production losses at both pre- and post-harvest stages [45,46,47,48]. The disease symptoms include dry brown necrotic spots, white mycelium, and water-soaked signs at the clove surface [47]. A previous study indicates that the *A. sativum PR5* (*AsPR5*) gene (AKU38392.1), which is upregulated by plant hormones methyl jasmonate (MeJA), abscisic acid (ABA), and ethylene, is also induced in response to *F. oxysporum* [49]. Given that in *Arabidopsis*, *PR5* expression confers resistance to a necrotrophic fungus *B. cinerea*, it can be suggested that *AsPR5* plays a similar role in garlic through regulation of signaling pathways associated with antifungal defense. The other three identified garlic TLP-coding genes, *AsPR5a* (Asa2G01043.1), *AsPR5b* (Asa4G02099.1), and *AsPR5c* (Asa4G02100.1) have been reported to be differentially expressed in the roots of FBR-resistant and -susceptible garlic cultivars in response to *F. proliferatum* attack [50].

Here, we identified in silico 32 putative *AsTLP* genes in the genome of *A. sativum* cultivar (cv.) Ershuizao, performed their structural characterization, analyzed phylogeny, and cloned *AsTLP* homologs from FBR-resistant and -susceptible garlic cultivars. Comparative expression profiling of *AsTLP1–32* in various tissues of these cultivars infected with *F. proliferatum* suggests a role of AsTLPs in the mechanisms underlying garlic defense against fungal pathogens. Our results provide new insights into the function of *A. sativum* TLPs, which can be used in breeding programs to increase the resistance to *Fusarium* in cultivated *Allium* spp.

## 2. Results

### 2.1. In Silico Genome-Wide Identification of TLP Genes in A. sativum cv. Ershuizao

**Figure 1:**
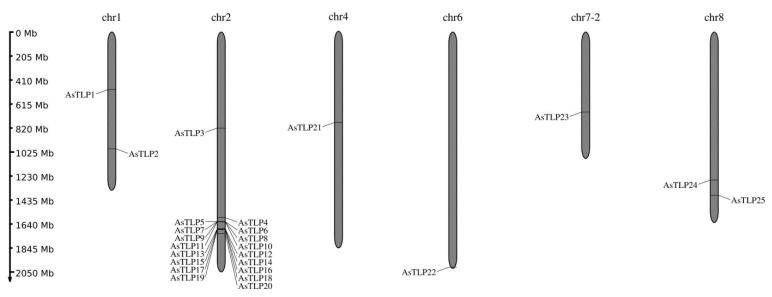
Locations of *AsTLP* genes in the *A. sativum* chromosomes. Chromosome lengths (indicated on the left) are based on the *A. sativum* cv. Ershuizao genome (PRJNA606385; assembly Garlic.V2.fa); chr, chromosome.

A total of 32 complete *TLP* gene sequences were detected in the genome and transcriptome of *A. sativum* cv. Ershuizao (PRJNA606385, PRJNA607255) and denoted *AsTLP1–32* (Table 1, Supplementary Table S1). Most of the genes (*AsTLP3–20*) are located on chromosome 2 and seven (*AsTLP1*, *2*, and *21–25*)—on chromosomes 1, 4, 6, 7, and 8 (Figure 1), whereas the other seven (*AsTLP26–32*) have been found in scaffolds and do not match any chromosomes (assembly Garlic.V2.fa; Table 1). The sizes of the identified genes range from 615 to 2572 bp and those of the coding sequences (CDSs)—from 615 to 972 bp (Table 1).

### 2.2. Structural and Phylogenetic Analyses of AsTLPs

**Figure 2:**
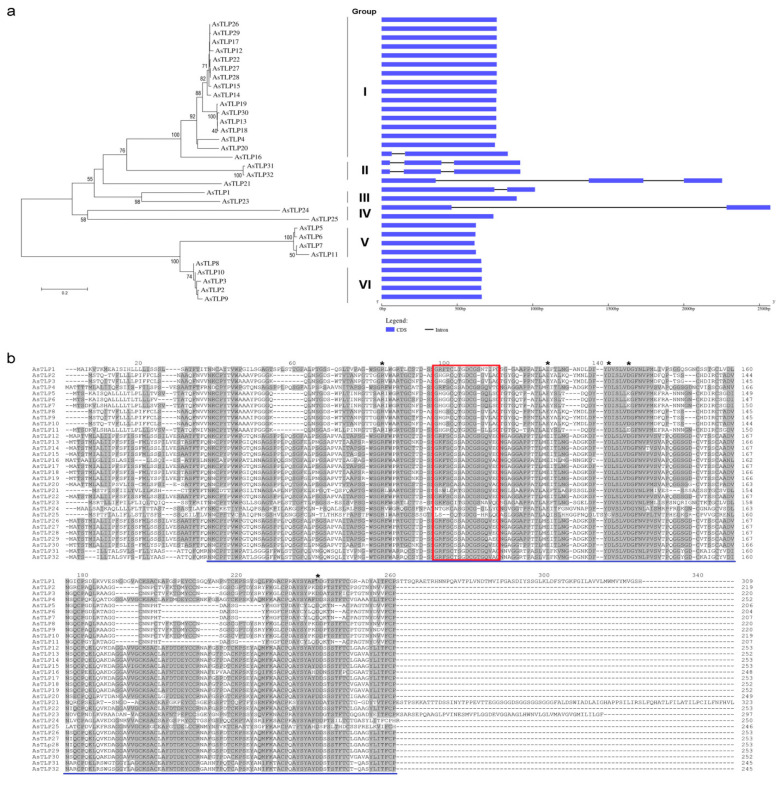
Phylogenetic and structural analysis of AsTLPs. (**a**) Evolutionary relationship based on amino acid sequences and exon-intron structures of the *AsTLP* genes. The unrooted dendrogram was constructed in MEGA 7.0.26 using the Neighbor-Joining method (bootstrap test: 1000 replicates). (**b**) Sequence alignment of AsTLPs. Regions with 50–100% identity are grey-shaded. The conserved thaumatin domain (GH64-TLP-SF; pfam00314) is underlined blue, the TLP family signature GX[GF]XCXT[GA]DCX(1,2)GX(2,3)C (PS00316) is framed red, and REDDD motif residues are marked with asterisks.

**Figure 3:**
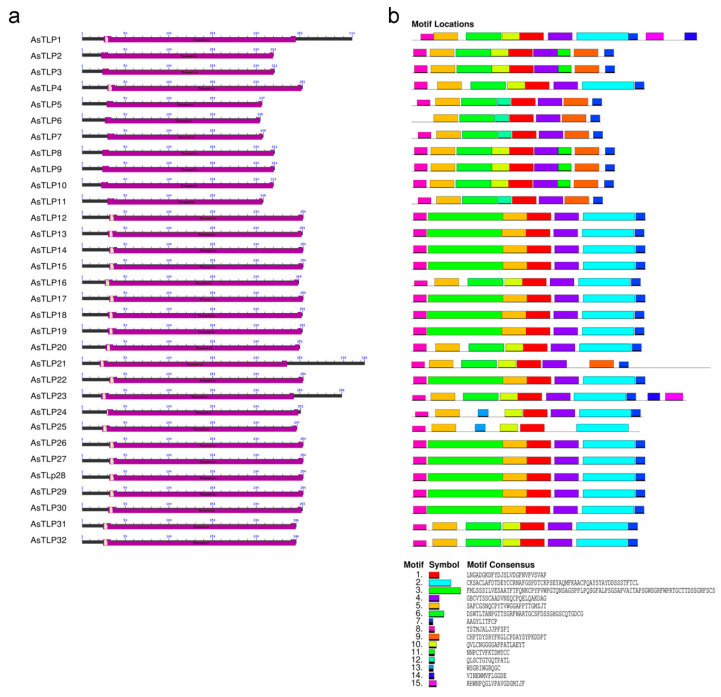
Position of the thaumatin domain (violet box) (**a**) and distribution of conserved motifs (**b**) in AsTLPs. Analysis was performed using MEME 5.3.0. The length of each box corresponds to that of the motif.

Most identified *AsTLP* genes (26) did not have introns, and the rest contained one (*AsTLP1*, *16*, *24*) or two (*AsTLP21*, *31*, *32*) introns (Figure 2a, Table 1). The characteristics of the translated proteins are shown in Table 1. Putative AsTLPs ranged in size from 204 to 323 amino acids (aa); among them, 13 were hydrophilic and 19-hydrophobic according to hydrophobicity indicators. All AsTLPs had similar structures, containing an N-terminal signal peptide (21–29 aa), full-length functional catalytic domain GH64-TLP-SF (glycoside hydrolase family 64 and TLP superfamily; pfam00314), thaumatin signature GX[GF]XCXT[GA]DCX(1,2)GX(2,3)C (PS00316), “REDDD” motif associated with antifungal activity, and 13–18 cysteine residues responsible for disulfide bond formation [44] (Figure 2b and Figure 3a, Table 1).

Phylogenetic analysis revealed six AsTLP clades (Figure 2A). The first and largest clade comprised 16 AsTLPs (73.4–99.6% identity), the second contained AsTLP31 and AsTLP32 (98.8% identity), the third contained AsTLP1 and AsTLP23 (63.9% identity), the fourth contained AsTLP24 and AsTLP25 (42.0% identity), the fifth containedAsTLP5 –7 and AsTLP11 (92.3–97.6% identity), and the sixth contained AsTLP2, AsTLP3, and AsTLP8–10 (95.9–99.1% identity). In the NCBI database of non-redundant protein sequences, AsTLPs of the last two clades show strong homology to *A. thaliana* osmotin 34 (ID: NP_192902.1) (Table 1).

The search for allergenic motifs disclosed statistically significant sequence similarity between AsTLPs and thaumatin-like allergens from apple (Mal d 2) [51], peach (Pru p 2.0101 and Pru p 2.0201) [52], cherry (Pru av 2) [31], and kiwi (Act c 2) [53].

In total, 15 conserved motifs were identified in putative AsTLPs, including motif 8 corresponding to signal peptide. MEME analysis indicated that phylogenetically related AsTLPs belonging to the same clade had similar conserved motifs (Figure 3b). Thus, motif 15 (consensus RHWNPQGLVPAVGDGMIJF) was detected only in clade III, motif 13 (WSGRIWGRQGC)—in clade IV, and motif 12 (QLSCTGTGQTPATL)—in clade V. The differences observed in the motif composition of the thaumatin domain corresponded to protein phylogeny (Figure 3b).

Annotation of AsTLPs in Gene Ontology (GO) terms predicted their extracellular localization (GO:0005576), as well as a role in defense response (GO:0006952) and response to fungal infection (GO:0009620).

### 2.3. In Silico Analysis of AsTLP Transcription

**Figure 4:**
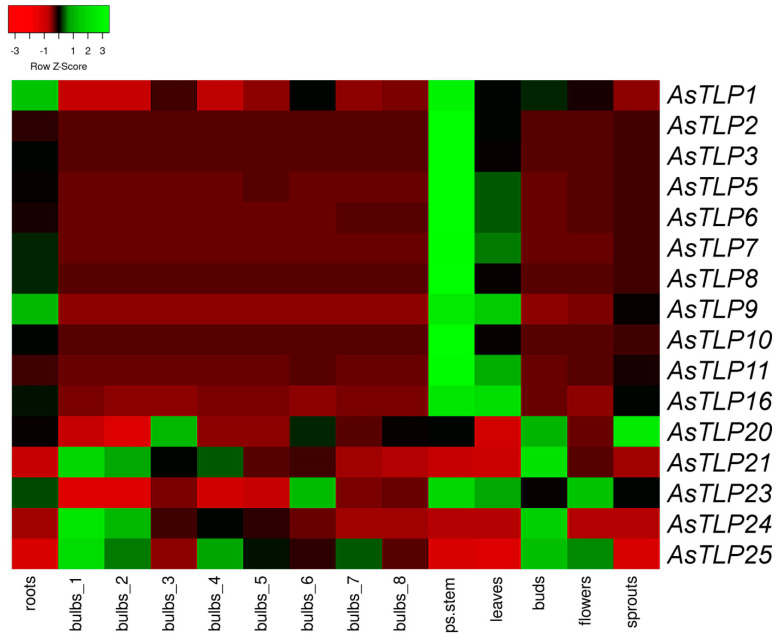
Heatmap of *AsTLP* gene expression in *A. sativum* tissues. Gene transcription was analyzed in the roots, bulbs (1, 2, 3, 4, 5, 6, 7, and 8 correspond to 192-, 197-, 202-, 207-, 212-;, 217-, 222-, and 227-day-old bulbs), stems (ps.stem), leaves, buds, flowers, and sprouts. The color gradient indicates expression changes from low (red) to high (green).

The expression of *AsTLP* genes in various tissues (roots, bulbs, stems, leaves, buds, flowers, and sprouts) of *A. sativum* cv. Ershuizao was evaluated based on transcriptomics data (PRJNA607255). As the bulb is the part of garlic most susceptible to *Fusarium* infection and shows visible symptoms, gene expression analysis in this organ was more detailed and included eight developmental stages (from day 192 to day 227 after planting). Figure 4 shows mRNA expression levels of 16 *AsTLP* genes, for which Fragments Per Kilobase of transcript per Million mapped reads (FPKM) was ≥ 10 in at least one of the organs. *AsTLP1–3*, *5–11*, and *16* had overall similar expression patterns (with the exception of *AsTLP1* and *9* in the roots and *AsTLP5-7*, *9*, *11*, and *16* in the leaves), whereas those of *AsTLP20*, *21*, and *23–25* were different.

About a third of the *AsTLP* genes (*AsTLP1–3*, *5–11*, and *16*) were strongly expressed in the stems and leaves. In the roots, the highest mRNA levels were observed for *AsTLP1*, *9*, and *23* and the lowest—for *AsTLP21*, *24*, and *25*; the latter had the highest expression in the floral buds and stage 1 bulbs, which gradually decreased during bulb development. The expression of *AsTLP20* and *23* in the bulbs showed bell-shape changes, with the maximum at stages 3 and 6, respectively. According to the expression profiles, the *AsTLP* genes could be divided into two groups: *AsTLP1–3*, *5–11*, and *16* with the highest expression in the stems, leaves, and roots, and *AsTLP20*, *21*, *24*, and *25* with that in the developing bulbs, floral buds, and flowers. The first group includes all genes of phylogenetic clades V and VI, as well as one gene each from clades I and III; members of the second group are phylogenetically heterogeneous (Figure 2a). The expression pattern of the *AsTLP23* gene had the features of both groups (Figure 4).

### 2.4. AsTLP Promoter Analysis

**Figure 5:**
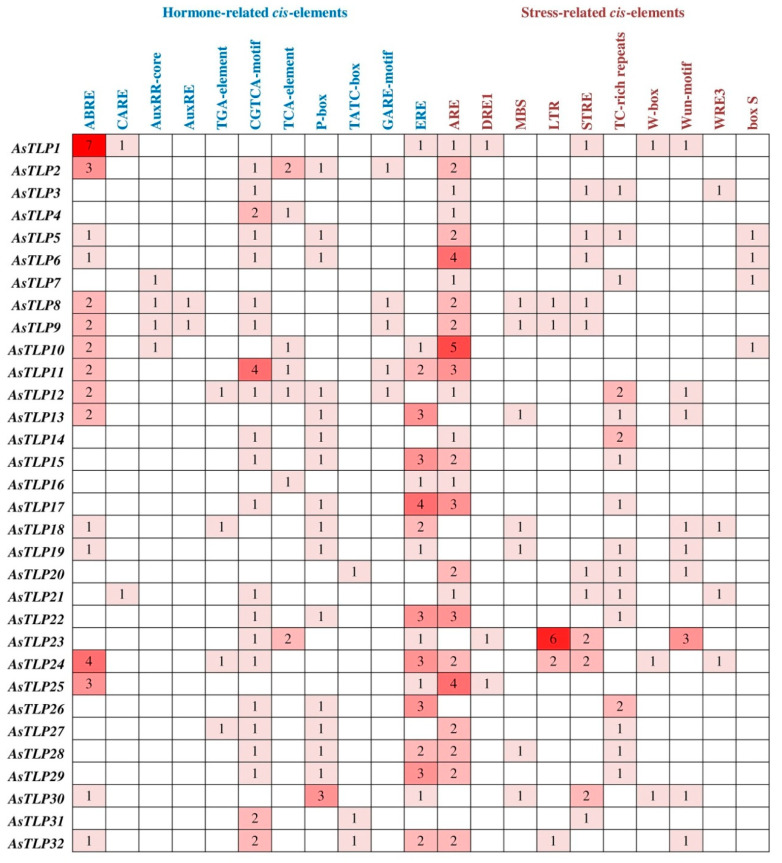
Hormone- and stress-responsive *cis*-elements in the regulatory regions (~1000 bp) of *AsTLP* genes. The color scheme (from pale to dark) corresponds to the numbers of *cis*-elements (from low to high).

Considering the stress- and hormone-responding role of TLPs [49], we searched for respective *cis*-acting elements in the 5′-UTR and promoter regions (1 kb upstream of the initiation codon) of *AsTLP* genes. The results indicated that the *AsTLP* regulatory regions contained 11 hormone-responsive and 10 stress-responsive elements (Figure 5). Among the former, the most common were the ABA-responsive element (ABRE) [54], ethylene-responsive element (ERE) critical for ethylene-regulated transcription in plants [55], a highly conserved prolamin-box (P-box) recognized by the trans-activator P-box-binding factor (PBF) during endosperm development [56], and the CGTCA motif involved in MeJA and osmotic stress responsiveness [57]. Among the stress-responsive elements, the most common were anaerobic-responsive element (ARE) involved in the activation of anaerobic gene expression [58] and TC-rich repeats found in promoters of many plant disease-resistance genes [59]. The promoters most enriched in the regulatory elements were those of *AsTLP11* (2 ABRE, 4 CGTCA motifs, 2 ERE, and 3 ARE), *AsTLP17* (4 ERE and 3 ARE), *AsTLP1*, *24*, and *25* (7, 4, and 3 ABRE, respectively), and *AsTLP6* and *10* (4 and 5 ARE, respectively). The promoters of five genes (*AsTLP8*, *9*, *23*, *24*, and *32*) contained low-temperature-responsive (LTR) elements; among them, *AsTLP23* had the highest number (6). The W-box, which responds to fungal elicitors and wounding, was found in only three genes (*AsTLP1*, *24*, and 30). The rarest elements were ABA-responsive CARE (in *AsTLP1* and *21*) and auxin-responsive AuxRE (in *AsTLP8* and *9*) (Figure 5). No pronounced similarity between the set of elements and the structure of genes was found, with the exception of some groups of genes, for example, *AsTLP5* and *6* (clade V), *8* and *9* (clade VI), or *22*, *28*, and *29* (clade I).

### 2.5. AsTLP1-32 Expression in cv. Sarmat and Strelets Infected with F. proliferatum

**Figure 6:**
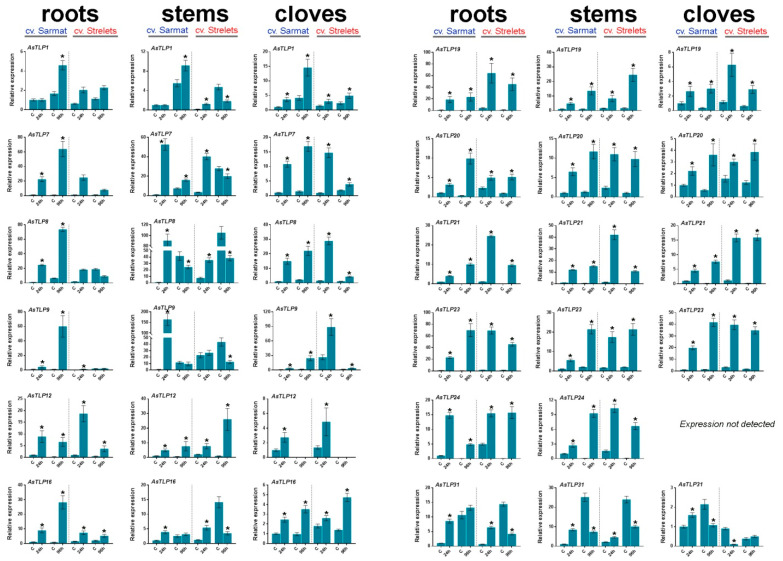
Expression of the *AsTLP* genes in the roots, stems, and cloves of *A. sativum* FBR-resistant cv. Sarmat and FBR-susceptible cv. Strelets at 24 and 96 hpi with *F. proliferatum*. *AsTLP8* represents the cumulative data for *AsTLP3*, *8* and *10*, *AsTLP12*—for *AsTLP12*, *15*, *17*, *22*, and *26–29*, and *AsTLP19*—for *AsTLP13*, *18*, *19*, and *30*. Transcription of *AsTLP5*, *6*, and *32* was not detected. The data were normalized to *GAPDH* and *UBQ* mRNA levels and presented as the mean ± SE (*n* = 3) compared to control (cv. Sarmat expression at 24 hpi taken as 1); * *p* < 0.01 compared to uninfected control.

**Figure 7:**
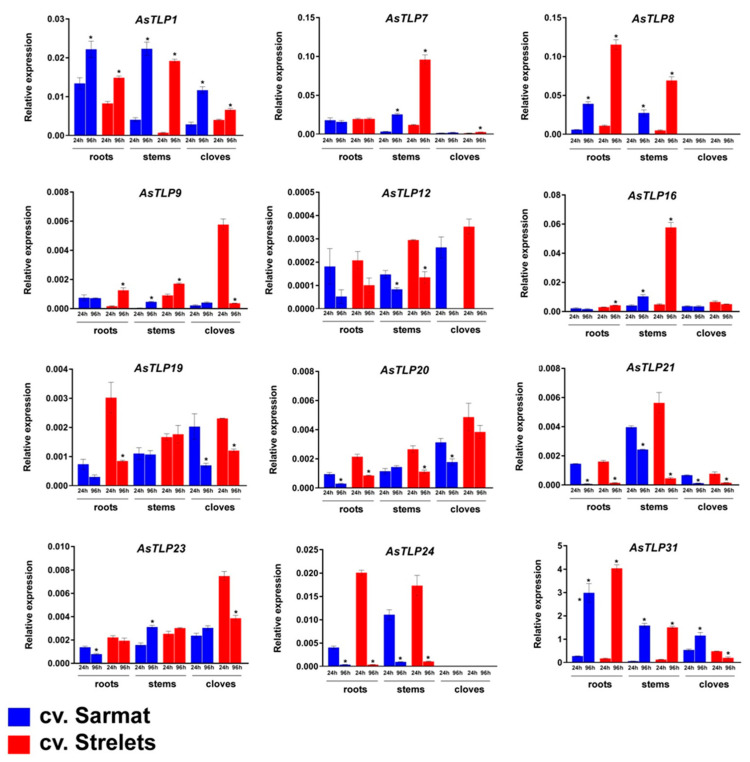
Time-dependent expression of *AsTLP* genes in the roots, stems (basal plates), and cloves of uninfected garlic cultivars resistant (cv. Sarmat) and susceptible (cv. Strelets) to FBR. The data were normalized to *GAPDH* and *UBQ* mRNA levels and presented as the mean ± SE (*n* = 3); * *p* < 0.01 indicates the difference between 24 and 96 h.

To elucidate the role of the identified *AsTLP1-32* genes in the garlic response to fungal infection, we performed *AsTLP* expression profiling in garlic cultivars resistant (cv. Sarmat) and susceptible (cv. Strelets) to FBR [60]. The *AsTLP7* corresponds to clade V group of genes (*AsTLP5*–*7* and *11*), *AsTLP8*—to set of *AsTLP3*, *8*, and *10* genes, *AsTLP12*—to set of *AsTLP12*, *15*, *17*, *22*, and *26-29* genes, *AsTLP19*—to *AsTLP13*, *18*, *19*, and *30* genes, and *AsTLP31*—to *AsTLP31* and *32* (clade II) genes (Supplementary Table S2). Gene expression was analyzed in the roots, stems (basal plates), and cloves of cv. Sarmat and cv. Strelets at 24 and 96 h post infection (hpi) with *F. proliferatum* and compared with uninfected control (Figure 6 and Figure 7).

The results revealed that the *AsTLP* genes were transcribed in all analyzed organs, except for *AsTLP24* not expressed in the cloves (Figure 6). To determine the expression of individual genes in clades V and II, we performed sequencing of PCR-amplified products, which revealed that only *AsTLP7* and *AsTLP31* were transcribed in the tissues of both cultivars. For the other groups of highly homologous *AsTLPs*, the amplified PCR products were invariable within each group.

In both uninfected cultivars, the highest expression was observed for *AsTLP7*, *8*, and *31* (Figure 7). Analysis of time-dependent changes revealed that most genes had similar expression dynamics in the two cultivars. Thus, the mRNA levels of *AsTLP1*, *7*, *8*, and *16* remained the same or increased from 24 to 96 hpi, whereas those of *AsTLP12*, *19–21*, and *24* were either the same or decreased. However, the expression of *AsTLP23* and *31* was increased from 24 to 96 hpi in the cloves of FBR-resistant cv. Sarmat but decreased in those of FBR-susceptible cv. Strelets (Figure 7).

Symptoms of *F. proliferatum* infection were observed only in FBR-sensitive cv. Strelets after 96 hpi: the roots were covered with white fungal mycelium, indicating the external growth of the pathogen. Infection changed the expression patterns of the *AsTLP* genes compared to uninfected control. Thus, in FBR-resistant cv. Sarmat, the transcription of all genes was upregulated at 24 hpi, except for that of *AsTLP1* in the roots and stems, which was upregulated at 96 hpi. These data suggest a correlation between the expression level of *AsTLP1* and the appearance of disease symptoms. In the roots, the expression of most genes was increased from 24 to 96 hpi, except for that of *AsTLP12* and *19* (unchanged) and *AsTLP24* (decreased). Similar dynamics were observed in the cloves, except for *AsTLP12* and *31*, which were downregulated with time. In the stems, the expression of *AsTLP7–9* and *31* decreased and that of *AsTLP19* and *24* increased from 24 to 96 hpi, whereas that of *AsTLP16* was unchanged (Figure 6).

In FBR-susceptible cv. Strelets, all *AsTLP* genes were upregulated at 24 hpi compared to uninfected control, except for *AsTLP9* in the roots and stem and *AsTLP31* in the cloves, which were upregulated at 96 hpi (Figure 6). The time-dependent *AsTLP* expression dynamics in cv. Strelets significantly differed from that in cv. Sarmat: the expression of *AsTLP7–9*, *20*, *21*, *23*, and *24* was either unchanged or downregulated from 24 to 96 hpi, whereas tissue-dependent changes were observed for the other genes. Thus, *AsTLP12* and *31* were downregulated with time in the roots and cloves and upregulated in the stems, *AsTLP16* was downregulated in the roots and stems and upregulated in the cloves, and *AsTLP19* was downregulated in the cloves and upregulated in the stems (Figure 6).

### 2.6. Cloning and Characterization of CDSs of AsTLP Genes Differentially Expressed in FBR-Sensitive and -Resistant Cultivars

Considering the differential expression of some *AsTLP* genes in cv. Sarmat and cv. Strelets in response to *F. proliferatum* infection (Figure 6), we investigated the association of these genes with FBR resistance. *AsTLP7*, *16*, and *20* were upregulated by the infection in the roots of cv. Sarmat, whereas *AsTLP21* and *23* were upregulated in those of cv. Strelets. The CDSs of these genes were amplified, cloned, and sequenced, and the data deposited in NCBI GenBank (Table 2).

The cv. Ershuizao *AsTLP* sequences were used as references. The amino acid sequences of AsTLP7, 16, 20, 21, and 23 from cv. Sarmat and Strelets did not differ in size from those of cv. Ershuizao AsTLPs. Compared to the cv. Ershuizao *AsTLP* genes, the cv. Sarmat and Strelets genes contained 4–18 single nucleotide polymorphisms (SNPs). Among them, non-synonymous SNPs leading to amino acid substitutions were found in *AsTLP7* (4), *AsTLP16* (1, resulting in radical A159G), *AsTLP20* (4, including radical G62R), *AsTLP21* (1), and *AsTLP23* (1) of both cultivars, whereas a cultivar-specific non-synonymous SNP (670T>C, leading to radical Y224H) was detected in *AsTLP20* of cv. Strelets (Table 2).

### 2.7. Analysis of Regulatory Regions in the AsTLP Genes Differentially Expressed in cv. Sarmat and Strelets after F. proliferatum Infection

**Figure 8:**
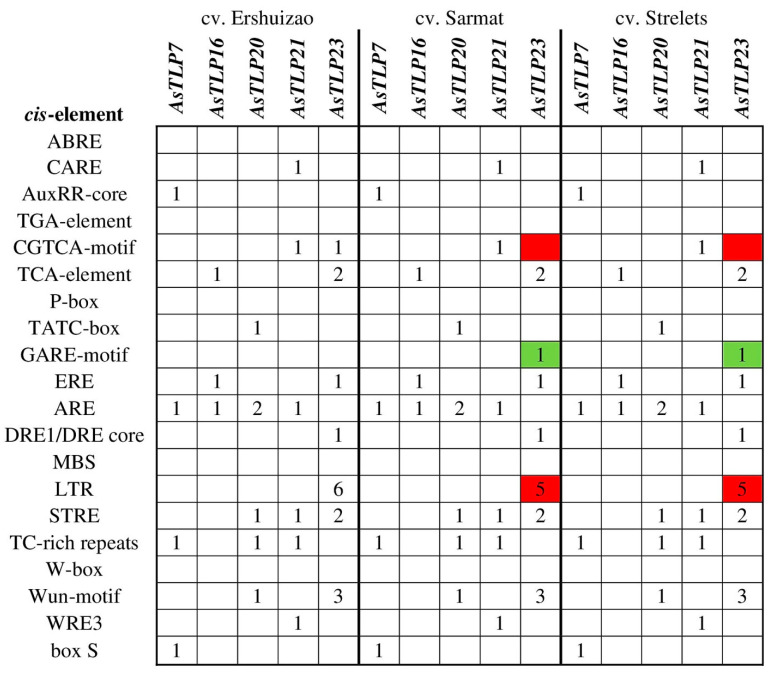
Comparative analysis of hormone- and stress-related *cis*-elements in the promoter regions of *AsTLP7*, *AsTLP16*, *AsTLP20*, *AsTLP21*, and *AsTLP23* genes in *A. sativum* cv. Ershuizao, cv. Sarmat, and cv. Strelets. The numbers of *cis*-elements are indicated. The elements present in cv. Sarmat and cv. Strelets but absent in cv. Ershuizao are highlighted green and those present in cv. Ershuizao but absent or less abundant in cv. Sarmat and cv. Strelets are highlighted red.

The regulatory sequences (promoter and 5′-UTR) of the *AsTLP7*, *16*, *20*, *21*, and *23* genes, which were differently expressed in cv. Sarmat and Strelets in response to *F. proliferatum* infection, were amplified, sequenced, and analyzed for hormone- and stress-responsive elements (Figure 8). The regulatory region of only the *AsTLP23* gene of cv. Sarmat and Strelets differed from the *AsTLP23* of cv. Ershuizao by the absence of the CGTCA-motif and one of the six LTR-elements (Figure 8). In general, the sequences of the regulatory regions in *AsTLP7*, *16*, *20*, *21*, and *23* were the same in cv. Sarmat and Strelets, but differed from those in cv. Ershuizao: there were 3 indels (11, 4, and 2 bp) and 20 SNPs in *AsTLP7*, 8 SNPs in *AsTLP20*, 3 indels (2, 2, and 7 bp) and 7 SNPs in *AsTLP21*, and 5 SNPs in *AsTLP23*.

The mutations found in the *AsTLP7*, *20*, and *21* regulatory sequences did not affect *cis*-elements, whereas those in the *AsTLP23* sequences did. Thus, two SNPs (at positions -674G>A and -676A>G) resulted in the loss of the CGTCA motif associated with MeJA response, another SNP (-521C>T) created the GARE motif (gibberellin-responsive element), and two SNPs (-640G>A and -641C>A) eliminated an LTR element (Figure 8).

## 3. Discussion

Garlic (*A. sativum* L.) belongs to the *Allium* genus, which comprises 971 species, thus being one of the largest genera of the Amaryllidaceae family widely distributed in the Northern Hemisphere from dry subtropics to boreal zones [61,62]. Significant garlic crop losses regularly occur worldwide, mostly because of diseases caused by soil fungi of *Fusarium* spp. [63,64], as well as by drought, osmotic, and cold stresses [35,65]. In many plants, TLPs play a critical role in the defense against both abiotic and biotic stresses [66], suggesting that they also perform a similar role in garlic and may potentially be used in *Allium* crop breeding programs.

In the present study, we identified and characterized 32 *TLP* genes in the *A. sativum* cv. Ershuizao genome [67] and amplified five of them (CDSs and 5′-regulatory regions) from garlic cv. Sarmat and Strelets differing in the susceptibility to FBR. All the genes encoded proteins homologous to the *A. thaliana* pathogenesis-related TLPs, including osmotin 34, implying the same functional activity. Among the identified *AsTLP* genes, eight were located in six chromosomes, where they probably have emerged because of local segmental duplications, and 17 were tandemly arranged in a single cluster in chromosome 2 (Figure 1), which may be due to tandem duplication through multiple episodes of unequal crossovers. This hypothesis is consistent with the clade distribution of *AsTLP* genes, when several tandemly duplicated genes were clustered together (Figure 2a), suggesting their origin from recent duplication events. Structure analysis revealed that most *AsTLPs* (26) had no introns or contained only a few (1–2) of them (Figure 2a). The intron-poor and intronless genes transcribed with less or without splicing are considered to have originated from intron-rich genes as a part of the adaptation strategy aimed to provide fast responses to different biotic and abiotic stresses, which is consistent with strong induction of intronless genes after stress [68].

All putative AsTLP1–32 proteins contained the conserved thaumatin domain and TLP family motif signatures (Figure 2b and Figure 3), suggesting conservation of garlic TLPs in terms of regulation through stress/defense-related signaling and functional activity to hydrolyze fungal β-1,3-glucan. The presence of N-terminal signal peptides indicates targeting of mature AsTLPs to the apoplastic pathway, which is supported by GO analysis predicting AsTLP extracellular secretion and is consistent with a previous report on TLP localization to the intercellular space [69]. These data agree well with the function of TLPs in hydrolyzing the fungal cell wall and their GO-predicted role in defense response to fungal infection.

All AsTLP1–32 proteins contained fragments with significant homology to previously described thaumatin-like allergens from various plant species [31,51,52,53]. Given the reports of IgE-mediated human hypersensitivity to raw garlic [70], it may be attributed, at least in part, to the AsTLPs expressed in the cloves (Figure 6).

Seven AsTLPs (AsTLP5–11) were found to be homologous to *A. thaliana* osmotin 34 (Table 1), which functions in the initiation of ABA responses, including the regulation of ABA-induced proline synthesis [10], suggesting the involvement of AsTLP5–11 in the garlic response to osmotic stress.

It is known that fungi-mediated biotic stress activates plant immune system through perception of pathogen-associated molecular patterns [71] with following formation of reactive oxygen species, induction of salicylic acid and jasmonic acid signaling, and upregulation of PR genes [72]. The promoters of all PR5 family *AsTLP* genes contained *cis*-regulatory elements associated with the activation of immune mechanisms through responses to stresses (anaerobic conditions, dehydration, low temperature, salinization, heavy metals, and wounding), elicitors, and hormones, (Figure 6). A similar set of elements has also been found in the promoters of *Gossypium barbadense* and *Rosa chinensis TLP* genes [73,74] and of the other garlic *PR1–5* genes [50,60]. Accordingly, the expression of *AsTLP* genes may be triggered by phytohormones (ethylene, salicylic acid, ABA, and MeJA), and stresses.

Ethylene and ABA homeostasis is known to be modulated in host plant in the response to fungal infection [60,75]. In accordance with this, the ERE and ABRE elements were found in the promoters of more than half of *AsTLPs* (Figure 5), which suggests these genes activation by ethylene and ABA after *Fusarium* attack.

Ethylene is known to crosstalk with ABA in response to abiotic stresses [75], such as low temperature (freezing), drought, and salinity, which cause osmotic stress and, consequently, activation of SnRK2 kinases as key signal transducers in the ABA pathway [76]. Considering the set of discovered stress responsive elements, it can be assumed that almost all *AsTLP* genes are involved in the response to osmotic stress, except for *AsTLP7*, *9*, *16*, and *20*, promoters of which do not contain neither the CGTCA motif, nor the DRE1, LTR, and MBS elements. Only five genes (*AsTLP8*, *9*, *23*, *24*, and *32*) may be involved in the response to cold stress (especially *AsTLP23*, which contains 5 LTR elements) (Figure 5).

Some *AsTLP* genes within the same clade (*AsTLP5*–*7*, *11* or *AsTLP2*, *3*, *8–10*) (Figure 2a) tended to be dissimilar in the promoter *cis*-regulatory motif patterns (Figure 5), suggesting that their transcription in response to adverse conditions might be differentially regulated.

Analysis of *TLP* genes with the established function in plant defense has predicted two unique combinations of regulatory elements to distinguish between abiotic and biotic stress responses: genes regulated by abiotic stresses contain the ABRE element, whereas those responding to fungal attack do not [77]. These data suggest that *AsTLP3*, *4*, *7*, *14–17*, *20–23*, *26–29*, and *31*, whose promoters do not have ABRE (Figure 5 and Figure 8), may be involved in the antifungal defense of garlic.

Among the five *AsTLP* genes amplified from FBR-resistant cv. Sarmat and FBR-susceptible cv. Strelets, only one contained a cultivar-specific SNP leading to an amino acid substitution (Table 2), suggesting that the antifungal resistance in garlic may not be attributed to *AsTLP* mutations, but rather to transcriptional and translational regulation of gene expression. This notion is supported by the differential expression of *AsTLP* genes in FBR-resistant and -susceptible cultivars infected with *F. proliferatum*. The most pronounced dissimilarity was the time-dependent upregulation of *AsTLP7–9* (homologs of *OSMOTIN 34*) and *AsTLP21* in the roots of cv. Sarmat and their downregulation in those of cv. Strelets (Figure 6), suggesting that the expression dynamics of these *AsTLP* genes may underlie the difference in antifungal resistance between the cultivars. Considering that the promoters of *AsTLP8* and *9* contain the ABRE element, whereas those of *AsTLP7* and *21* do not, the latter are more likely to have a role in the garlic defense responses against *F. proliferatum*.

It has been suggested that *TLP* genes can serve as molecular markers associated with resistance to fungal diseases [69]. Thus, our results could be useful for breeding programs aimed on increasing the resistance of garlic crops to *Fusarium* spp. by, for example, generating cultivars that overexpress the respective *AsTLP* genes, which may protect against fungal infections. In addition, it should be noted that, besides TLPs, the fungicidal effects of *Allium* plants are associated with chitinases and endo-1,3-β-glucanases, as well as miRNAs involved in positive (miR164a, miR168a, and miR393) and negative (miR394) regulation of resistance to *Fusarium* [48,50,60,78]. Moreover, *Allium* roots produce antifungal volatiles such as 2-methyl-2-pentenal and organosulfur compounds, as well as non-volatiles such as spirostanol, furostanol, and steroidal saponins, which inhibit *Fusarium* growth [60,79,80].

## 4. Materials and Methods

### 4.1. In Silico Identification and Structural Characterization of AsTLP Genes

The search for *TLP* genes was performed in the *A. sativum* cv. Ershuizao whole-genome (NCBI accession number: PRJNA606385, assembly Garlic.V2.fa) and transcriptome (PRJNA607255) sequences [68]. The thaumatin domain (http://pfam.xfam.org/family/PF00314; accessed on 25 October 2021) was used as reference. All the selected sequences contained start and stop codons and full-length catalytic domains.

Multiple sequence alignment and structural analyses of the *AsTLP* genes and encoded proteins were conducted with MEGA 7.0.26 [81]. The phylogenetic dendrogram was constructed based on protein sequences using the MEGA 7.0.26 (ML method); confidence for tree topologies was estimated by bootstrap values of 1000 replicates.

To predict exon–intron composition, *AsTLP* genes and CDSs were analyzed with GSDS v2.0 [82]. Putative proteins were characterized by molecular weight, pI, and grand average hydropathy (GRAVY) (ExPASy ProtParam; https://web.expasy.org/protparam/; accessed on 30 October 2021; GRAVY indexes were calculated as the sum of hydrophobicity values of all residues divided by sequence length), conserved domains, sites, and motifs (NCBI-CDD, https://www.ncbi.nlm.nih.gov/cdd; accessed on 30 October 2021; and Multiple Em for Motif Elicitation (MEME 5.3.0), http://meme-suite.org/tools/meme; accessed on 30 October 2021), biological processes (PANNZER2; http://ekhidna2.biocenter.helsinki.fi/sanspanz/; accessed on 30 October 2021), subcellular localization (BaCello; http://gpcr2.biocomp.unibo.it/; accessed on 30 October 2021), functional importance of residue substitutions (PROVEAN; http://provean.jcvi.org/seq_submit.php; accessed on 25 November 2021), and signal peptide cleavage sites (SignalP 5.0; http://www.cbs.dtu.dk/services/SignalP/; accessed on 30 October 2021). The chromosomal localization map was drawn using MG2C v. 2.1 (http://mg2c.iask.in/mg2c_v2.1/; accessed on 30 October 2021). The search for allergenic motifs in AsTLPs was conducted using the Structural Database of Allergenic Proteins (SDAP) (https://fermi.utmb.edu/SDAP/sdap_fas.html; accessed on 19 January 2022).

### 4.2. In Silico mRNA Expression Analysis

The expression of *AsTLP* genes in the roots, bulbs, stems (basal plates), leaves, buds, flowers, and sprouts was analyzed based on *A. sativum* cv. Ershuizao RNA-Seq data (FPKM; ID: PRJNA607255), normalized as FPKM [66], and visualized using Heatmapper [83]. Only transcripts with an average FPKM value ≥ 10 in at least one of the organs were used for heatmap construction.

### 4.3. Gene Identification

To amplify the *AsTLP* CDSs from garlic cultivars, gene-specific primers were designed based on *A. sativum* cv. Ershuizao transcriptomic data (NCBI project accession number: PRJNA607255) (Supplementary Table S2). CDNA (30 ng) from the roots of each cultivar accession was used as a template for PCR amplification at the following conditions: initial denaturation at 95 °C for 5 min, 35 cycles [denaturation at 95 °C for 30 s, primer annealing at 55 °C for 30 s, and extension at 72 °C for 2 min], and final extension at 72 °C for 5 min. PCR products of the expected size were purified by using the QIAEX^®^ II Gel Extraction kit (QIAGEN, Hilden, Germany), cloned in the pGEM^®^-T Easy vector (Promega, Madison, WI, USA), and sequenced (3–5 clones for each accession) on ABI Prism 3730 DNA Sequencer (Applied Biosystems, Waltham, MA, USA) using the designed primers.

### 4.4. Plants, Fungi, and F. proliferatum Infection Assay

*F. proliferatum* was kindly provided by the Group of Experimental Mycology, Winogradsky Institute of Microbiology (Research Center of Biotechnology of the RAS, Moscow, Russia). The strain was originally isolated from the bulbs of garlic cv. Strelets; according to the pathogenicity test, the first signs of the disease appeared on the clove surface 5 days after infection [50].

Accessions of *A. sativum* cv. Sarmat and cv. Strelets (winter garlic of Russian breeding) resistant and susceptible to FBR, respectively, were kindly provided by the Federal Scientific Vegetable Center (Moscow region, Russia). The number of cloves used per biological replicate in the *Fusarium* infection assay was based on that of cloves in the bulb (5–7 for cv. Strelets and 7–11 for cv. Sarmat). In total, 12 cloves of each cultivar (6 infected and 6 uninfected) were processed (three biological replicates were used). Cloves were surface-sterilized in 70% ethanol for 3 min, rinsed with sterile water, placed in Petri dishes with wet filter paper, and incubated at +25 °C in the dark. After 72 h, active root growth was observed, and half of the cloves were infected by soaking in *F. proliferatum* conidial suspension (~10^6^ conidia ml^−1^) for 5 min as previously described [60]), transferred to fresh Petri dishes, and incubated at +25 °C in the dark for 24 and 96 h (*n* = 3 cloves per each time point).The roots, stems, and cloves of the infected and uninfected samples were collected at each time point, frozen in liquid nitrogen, and stored at −80 °C. The time points were chosen according to the expression peaks of some PR genes, which were observed 1–3 days after inoculation with hemibiotrophic pathogens [84].

### 4.5. RNA Extraction and Quantitative Real-Time Reverse Transcription PCR (qRT-PCR)

Total RNA was extracted from individual roots, stems, and cloves (0.5 g of each tissue) using the RNeasy Plant Mini Kit (QIAGEN, Hilden, Germany), purified from genomic DNA (RNase free DNase set; QIAGEN), qualified by gel electrophoresis, and used for first-strand cDNA synthesis (GoScript Reverse Transcription System; Promega, Madison, USA) with an oligo-dT primer. RNA and cDNA concentrations were quantified by fluorimetry (Qubit^®^ Fluorometer, Thermo Fisher Scientific, Waltham, MA, USA) and qRT-PCR was performed in a CFX96 Real-Time PCR Detection System (Bio-Rad Laboratories, Hercules, CA, USA) with 3.0 ng cDNA, SYBR Green RT-PCR mixture (Syntol, Moscow, Russia), and specific primers (Supplementary Table S2). Because CDSs of some AsTLPs had a high degree of homology, universal primers were designed for such genes, which were grouped according to their homology (Supplementary Table S2). The following cycling conditions were used: initial denaturation at 95 °C for 5 min, 40 cycles of denaturation at 95 °C for 15 s, and annealing/extension at 60 °C for 40 s.

*AsTLP* gene expression was normalized using two reference garlic genes, *GAPDH* and *UBQ* [60], and the qRT-PCR results were statistically analyzed with Graph Pad Prism version 8 (GraphPad Software Inc., San Diego, CA, USA; https://www.graphpad.com/scientific-software/prism/ (accessed on 30 October 2021)). The data were expressed as the mean ± standard deviation (SE) based on three technical replicates of three biological replicates for each combination of cDNA and primer pairs. The unequal variance (Welch’s) *t*-test was applied to assess differences in gene expression; *p* < 0.01 was considered to indicate statistical significance.

### 4.6. Promoter and 5′-UTR Analysis

The search for specific *cis*-elements in the promoters and 5′-UTRs (1.0 kb regions upstream of the initiation codon) was performed using the PlantCARE database, which provides evaluation of *cis*-regulatory elements, enhancers, and repressors; (http://bioinformatics.psb.ugent.be/webtools/plantcare/html/; accessed on 25 November 2021).

## 5. Conclusions

We identified and characterized 32 genes encoding thaumatin-like proteins in *A. sativum* cv. Ershuizao genome. The *AsTLP* genes were distributed among six chromosomes and four scaffolds and might have been evolutionary originated from segmental or tandem duplications. *AsTLP7*, *16*, *20*, *21*, and *23* homologs were amplified from garlic cultivars resistant and susceptible to *Fusarium* infection. The promoters of *AsTLP* genes contained distinct sets of *cis*-acting elements associated with hormone and stress reactivity, suggesting differential transcriptional regulation of garlic TLPs in response to pathogens and abiotic stresses, which was consistent with specific expression patterns of *AsTLP* genes in garlic cultivars infected with *F. proliferatum*. The transcription of *AsTLP7*–*9*, and *21* genes in the roots was downregulated in FBR-susceptible and upregulated in FBR-resistant cultivars, suggesting their particular involvement in the sensitivity of garlic to fungal infection. Our results provide the foundation for further functional characterization of the *AsTLP* genes using a reverse genetics strategy, and may contribute in the breeding of *A. sativum* cultivars with increased resistance to *Fusarium* infections as well as various abiotic stresses.

## Figures and Tables

**Table 1:** Characteristics of the predicted *TLP* genes in the *A. sativum* cv. Ershuizao genome.

Gene	Genomic Location (Strand)	*A. thaliana* Homolog	Transcript ID in RNA-Seq Database	Size (bp)	Exons	CDS (bp)	Protein					
							Size (aa)	MW (kDa)	pI	Signal Peptide	GH64 Domain	GRAVY
*AsTLP1*	Chr1:491780133..491781147 (+)	Pathogenesis-related thaumatin superfamily protein NP_001031792.1	Asa2G02997.1	1013	2	930	309	32.29	4.93	1–24	31–245	0.126
*AsTLP2*	Chr1:996536859..996536201 (−)	Osmotin 34 NP_192902.1	Asa2G01042.1	660	1	660	219	23.46	5.52	1–20	23–219	−0.157
*AsTLP3* (*AsPR5a*)	Chr2:821219552..821220214 (−)		Asa2G01043.1	663	1	663	220	23.46	4.71	1–21	24–220	−0.085
*AsTLP4*	Chr2:1584165307..1584166065 (−)	TLP AAD02499.1	Asa3G06018.1	759	1	759	252	25.92	4.79	1–27	30–251	0.069
*AsTLP5*	Chr2:1617805168..1617805788 (−)	Osmotin 34 NP_192902.1	Asa4G02103.1 Asa4G02102.1	621	1	621	206	21.89	4.76	1–26	29–206	−0.289
*AsTLP6*	Chr2:1617847011..1617847625 (−)			615	1	615	204	21.74	4.76	1–24	27–204	−0.263
*AsTLP7*	Chr2:1617850920..1617851543 (−)		Asa4G02101.1	624	1	624	207	22.02	4.77	1–27	30–207	−0.295
*AsTLP8* (*AsPR5b*)	Chr2:1618362333..1618362995 (+)		Asa4G02099.1	663	1	663	220	23.50	4.71	1–21	24–220	−0.126
*AsTLP9* (*AsPR5c*)	Chr2:1618377059..1618377721 (+)		Asa4G02100.1	663	1	663	220	23.59	4.74	1–21	24–220	−0.164
*AsTLP10*	Chr2:1618405507..1618406165 (−)		Asa3G05858.1	660	1	660	219	23.43	4.71	1–20	23–219	−0.126
*AsTLP11*	Chr2:1622416758..1622417381 (−)		Asa3G05840.1	624	1	624	207	22.08	5.70	1–27	30–207	−0.211
*AsTLP12*	Chr2:1684781475..1684782236 (+)	TLP AAD02499.1	Asa3G06302.1	762	1	762	253	26.16	4.27	1–29	32–252	0.056
*AsTLP13*	Chr2:1685003219..1685003978 (−)		Asa3G06331.1	759	1	759	252	26.28	4.24	1–28	31–251	0.012
*AsTLP14*	Chr2:1685049653..1685050414 (−)		no detected	762	1	762	253	26.06	4.18	1–29	32–252	0.087
*AsTLP15*	Chr2:1685078657..1685079418 (−)		no detected	762	1	762	253	26.06	4.18	1–29	32–252	0.068
*AsTLP16*	Chr2:1685746745..1685747579 (+)		Asa3G06310.1	835	2	747	248	25.30	4.59	1–25	27–247	0.031
*AsTLP17*	Chr2:1686634047..1686634808 (+)	Pathogenesis-related thaumatin superfamily protein NP_173432.2	Asa3G06319.1	762	1	762	253	26.09	4.18	1–29	32–252	0.054
*AsTLP18*	Chr2:1687188296..1687189055 (−)	TLP AAD02499.1	Asa3G06331.1	759	1	759	252	26.30	4.24	1–28	31–251	0.013
*AsTLP19*	Chr2:1687209664..1687210422 (−)		Asa3G06329.1	759	1	759	252	26.21	4.27	1–28	31–251	0.025
*AsTLP20*	Chr2:1724629365..1724630114 (+)	Pathogenesis-related thaumatin superfamily protein NP_973870.1	Asa7G02264.1	750	1	750	249	25.42	4.39	1–26	28–248	0.081
*AsTLP21*	Chr4:777618126..777620378 (+)	Pathogenesis-related thaumatin superfamily protein NP_568046.1	Asa4G00901.1	2253	3	972	323	33.86	4.50	1–19	26–232	−0.008
*AsTLP22*	Chr6:2008642583..2008643344 (+)	TLP AAD02499.1	Asa3G06325.1	762	1	762	253	26.09	4.18	1–29	32–252	0.085
*AsTLP23*	Chr7:682376071..682376964 (+)	Pathogenesis-related thaumatin superfamily protein NP_001119140.1	Asa5G05974.1	894	1	894	297	31.13	4.68	1–21	23–242	0.115
*AsTLP24*	Chr8:1265530915..1265533486 (−)	Pathogenesis-related thaumatin superfamily protein NP_177503.1	Asa7G04908.1	2572	2	753	250	25.97	9.07	1–26	31–247	0.081
*AsTLP25*	Chr8:1394582992..1394583731 (+)	Pathogenesis-related thaumatin superfamily protein NP_001324474.1	Asa7G04378.1	741	1	741	246	26.03	8.10	1–21	24–245	−0.005
*AsTLP26*	Scaffold9088: 51512..52273	Pathogenesis-related thaumatin superfamily protein NP_173432.2	Asa3G06323.1	762	1	762	253	26.09	4.18	1–29	32–252	0.054
*AsTLP27*	Scaffold9089: 39596..38835	TLP AAD02499.1	Asa3G06322.1	762	1	762	253	26.12	4.18	1–29	32–252	0.075
*AsTLP28*	Scaffold9089: 40346..41107			762	1	762	253	26.12	4.18	1–29	32–252	0.075
*AsTLP29*	Scaffold9089: 89114..89875		Asa3G06320.1	762	1	762	253	26.06	4.18	1–29	32–252	0.044
*AsTLP30*	Scaffold9091: 149728..150486		Asa3G06315.1	759	1	759	252	26.28	4.24	1–28	31–251	0.006
*AsTLP31*	Scaffold12619: 87332..88248		no detected	917	3	738	245	26.47	5.75	1–22	25–244	−0.134
*AsTLP32*	Scaffold12619: 99641..100558		no detected	918	3	738	245	26.46	5.75	1–22	25–244	−0.149

**Table 2:** Polymorphisms in the *AsTLP* CDSs of cv. Sarmat and Strelets compared to cv. Ershuizao.

Gene	NCBI ID cv. Sarmat/cv. Strelets	cv. Sarmat	cv. Strelets
		SNPs (aa Substitution)	
*AsTLP7*	OM386716/OM386717	c.183A>C, ****c.218G>C (p.G73A)**, c.219A>C (p.G73A)**, c.225A>C, **c.233A>G (p.N78S)**, c.243G>C, c.249G>A, c.252T>C, c.276T>C, c.279T>C, c.303A>C, c.333T>C, c.357T>C, c.378C>G, c.384C>T, c.399 >T, **c.479>T (p.T160M),** c.615C>T	
*AsTLP16*	OM386718/OM386719	c.141T>C, c.162T>C, c.406C>T, **c.476C>G (p. A159G)**	
*AsTLP20*	OM386720/OM386721	**c.49T>C (p.S17P)**, **c.53G>T (p.C18F)**, **c.67T>C (p.S23P)**, c.70A>G, c.75A>T, **c.184G>C (p.G62R)**	
			**c. 670T>C (p.Y224H)**
*AsTLP21*	OM386722/OM386723	c.22A>G, c.141G>A, c.453A>G, c.554G>A, c.570C>T, c.573T>G, c.738A>G, c.804A>T, **c.929C>G (p.A310G)**	
*AsTLP23*	OM386724/OM386725	c.T30>C, **c.A45>T (p.Q15H)**, c.A99>G, c.T147>G, c.C165>G, c.C588>G, c.C684>T, **c.C821>G (p. A274G)**, c.C847>T, c.C849>T, c.T851>C	

Note: Non-synonymous SNPs and corresponding amino acid substitutions are marked in bold.

## Data Availability

*AsTLP* CDSs of *A. sativum* cv. Sarmat/cv. Strelets were deposited in NCBI (see Table 2).

## Supplementary Materials

The following supporting information can be downloaded at: https://www.mdpi.com/article/10.3390/plants11060748/s1, Table S1: *AsTLP* CDSs and the corresponding amino acid sequences. Table S2: List of primers for *AsTLP* gene amplification, sequencing, and expression analysis.

## References

[1] Selitrennikoff C.P. (2001). Antifungal proteins. Appl. Environ. Microbiol..

[2] Vander Wel H., Loeve K. (1972). Isolation and characterization of thaumatin I & II, the sweet-tasting proteins from *Thaumatoccus danielli*. Eur. J. Biochem..

[3] De Jesús-Pires C., Ferreira-Neto J.R.C., Pacifico Bezerra-Neto J., Kido E.A., de Oliveira Silva R.L., Pandolfi V., Wanderley-Nogueira A.C., Binneck E., da Costa A.F., Pio-Ribeiro G. (2020). Plant Thaumatin-like Proteins: Function, Evolution and Biotechnological Applications. Curr. Protein Pept. Sci..

[4] Vitali A., Pacini L., Bordi E., De Mori P., Pucillo L., Maras B., Botta B., Brancaccio A., Giardina B. (2006). Purification and characterization of an antifungal thaumatin-like protein from *Cassia didymobotrya* cell culture. Plant Physiol. Biochem..

[5] Chowdhury S., Basu A., Kundu S. (2015). Cloning, characterization, and bacterial over-expression of an osmotin-like protein gene from *Solanum nigrum* L. with antifungal activity against three necrotrophic fungi. Mol. Biotechnol..

[6] Saeidi M., Zareie R. (2020). Prediction, isolation, overexpression and antifungal activity analysis of *Medicago truncatula* var. *truncatula* putative thaumatin like proteins (TLP-1, -2, -3, -4 and -5). Turk. J. Biol..

[7] Mahmood T., Jan A., Kakishima M., Komatsu S. (2006). Proteomic analysis of bacterial-blight defense-responsive proteins in rice leaf blades. Proteomics.

[8] Rodrigo I., Vera P., Frank R., Conejero V. (1991). Identification of the viroid-induced tomato pathogenesis-related (PR) protein P23 as the thaumatin-like tomato protein NP24 associated with osmotic stress. Plant Mol. Biol..

[9] Looze Y., Boussard P., Huet J., Vandenbusche G., Azarkan M., Raussens V., Wintjens R. (2009). Purification and characterization of a wound-inducible thaumatin-like protein from the latex of *Carica papaya*. Phytochemistry.

[10] Park E.J., Kim T.H. (2021). Thaumatin-like genes function in the control of both biotic stress signaling and ABA signaling pathways. Biochem. Biophys. Res. Commun..

[11] Wang X., Tang C., Deng L., Cai G., Liu X., Liu B., Han Q., Buchenauer H., Wei G., Han D. (2010). Characterization of a pathogenesis-related thaumatin-like protein gene TaPR5 from wheat induced by stripe rust fungus. Physiol. Plant..

[12] He L., Li L., Zhu Y., Pan Y., Zhang X., Han X., Li M., Chen C., Li H., Wang C. (2021). BolTLP1, a Thaumatin-like Protein Gene, Confers Tolerance to Salt and Drought Stresses in Broccoli (*Brassica oleracea* L. var. *Italica*). Int. J. Mol. Sci..

[13] Pluskota W.E., Pupel P., Głowacka K., Okorska S.B., Jerzmanowski A., Nonogaki H., Górecki R.J. (2019). Jasmonic acid and ethylene are involved in the accumulation of osmotin in germinating tomato seeds. J. Plant Physiol..

[14] Futamura N., Tani N., Tsumura Y., Nakajima N., Sakaguchi M., Shinohara K. (2006). Characterization of genes for novel thaumatin-like proteins in *Cryptomeria japonica*. Tree Physiol..

[15] An M., Tong Z., Ding C., Wang Z., Sun H., Xia Z., Qi M., Wu Y., Liang Y. (2019). Molecular Characterization of the Thaumatin-like Protein PR-NP24 in Tomato Fruits. J. Agric. Food Chem..

[16] Chen W.P., Chen P.D., Liu D.J., Kynast R., Friebe B., Velazhahan R., Muthukrishnan S., Gill B.S. (1999). Development of wheat scab symptoms is delayed in transgenic wheat plants that constitutively express a rice thaumatin-like protein gene. Theor. Appl. Genet..

[17] Velazhahan R., Muthu Krishnan S. (2003). Transgenic tobacco plants constitutively overexpressing a rice thaumatin-like protein (PR-5) show enhanced résistance to *Alternaria alternate*. Biol. Plant..

[18] El-kereamy A., El-sharkawy I., Ramamoorthy R., Taheri A., Errampalli D., Kumar P., Jayasankar S. (2011). *Prunus domestica* pathogenesis-related protein-5 activates the defense response pathway and enhances the resistance to fungal infection. PLoS ONE.

[19] Acharya K., Pal A.K., Gulati A., Kumar S., Singh A.K., Ahuja P.S. (2013). Overexpression of *Camellia sinensis* thaumatin-like protein, CsTLP in potato confers enhanced resistance to *Macrophomina phaseolina* and *Phytophthora infestans* infection. Mol. Biotechnol..

[20] He R., Wu J., Zhang Y., Agüero C.B., Li X., Liu S., Wang C., Walker M.A., Lu J. (2017). Overexpression of a thaumatin-like protein gene from *Vitis amurensis* improves downy mildew resistance in *Vitis vinifera* grapevine. Protoplasma.

[21] Odeny Ojola P., Nyaboga E.N., Njiru P.N., Orinda G. (2018). Overexpression of rice thaumatin-like protein (Ostlp) gene in transgenic cassava results in enhanced tolerance to *Colletotrichum gloeosporioides* f. sp. *manihotis*. J. Genet. Eng. Biotechnol..

[22] Wardhan V., Pandey A., Chakraborty S., Chakraborty N. (2016). Chickpea transcription factor CaTLP1 interacts with protein kinases, modulates ROS accumulation and promotes ABA-mediated stomatal closure. Sci. Rep..

[23] Singh N.K., Kumar K.R.R., Kumar D., Shukla P., Kirti P.B. (2013). Characterization of a pathogen induced thaumatin-like protein gene *AdTLP* from *Arachis diogoi*, a wild peanut. PLoS ONE.

[24] Munis M.F., Tu L., Deng F., Tan J., Xu L. (2010). A thaumatin-like protein gene involved in cotton fiber secondary cell wall development enhances resistance against *Verticillium dahliae* and other stresses in transgenic tobacco. Biochem. Biophys. Res. Commun..

[25] Anand A., Lei Z., Sumner L., Mysore K., Arakane Y. (2004). Apoplastic extracts from a transgenic wheat line exhibiting lesion-mimic phenotype have multiple pathogenesis-related proteins that are antifungal. Mol. Plant Microbe Interact..

[26] Grenier J., Potvin C., Asselin A. (1993). Barley pathogenesis-related proteins with fungal cell wall lytic activity inhibit the growth of yeasts. Plant Physiol..

[27] Mitchum M.G., Wang X., Wang J., Davis E.L. (2012). Role of nematode peptides and other small molecules in plant parasitism. Annu. Rev. Phytopathol..

[28] Kirino H., Yoshimoto K., Shinya R. (2020). Thaumatin-like proteins and a cysteine protease inhibitor secreted by the pine wood nematode *Bursaphelenchus xylophilus* induce cell death in *Nicotiana benthamiana*. PLoS ONE.

[29] Shinya R., Morisaka H., Kikuchi T., Takeuchi Y., Ueda M., Futai K. (2013). Secretome Analysis of the Pine Wood Nematode *Bursaphelenchus xylophilus* Reveals the Tangled Roots of Parasitism and Its Potential for Molecular Mimicry. PLoS ONE.

[30] Breiteneder H. (2004). Thaumatin-like proteins—A new family of pollen and fruit allergens. Allergy.

[31] Izumi E., Hidaka S., Hiroi A., Kinugasa S., Yano E., Zaima N., Moriyama T. (2021). Thaumatin-Like Protein (Pru av 2) Is a Cherry Allergen That Triggers Percutaneous Sensitization in Mice. Foods.

[32] Ghosh R., Chakrabarti C. (2008). Crystal structure analysis of NP24-I: A thaumatin-like protein. Planta.

[33] Sharma A., Shumayla, Tyagi S., Alok A., Singh K., Upadhyay S.K. (2020). Thaumatin-like protein kinases: Molecular characterization and transcriptional profiling in five cereal crops. Plant Sci..

[34] Chan Y.W., Tung W.L., Griffith M., Chow K.C. (1999). Cloning of a cDNA encoding the thaumatin-like protein of winter rye (*Secale cereale* L. Musketeer) and its functional characterization. J. Exp. Bot..

[35] Liu J.J., Sturrock R., Ekramoddoullah A.K.M. (2010). The superfamily of thaumatin-like proteins: Its origin, evolution, and expression towards biological function. Plant Cell Rep..

[36] Petre B., Major I., Rouhier N., Duplessis S. (2011). Genome-wide analysis of eukaryote thaumatin-like proteins (TLPs) with an emphasis on poplar. BMC Plant Biol..

[37] Shiu S.H., Bleecker A.B. (2001). Plant receptor-like kinase Gene family: Diversity, function, and signaling. Sci. Signal..

[38] Wang X., Zafian P., Choudhary M., Lawton M. (1996). The PR5K receptor protein kinase from *Arabidopsis thaliana* is structurally related to a family of plant defense proteins. Proc. Natl. Acad. Sci. USA.

[39] Guo Z., Bonos S., Meyer W.A., Day P.R., Belanger F.C. (2003). Transgenic creeping bentgrass with delayed dollar spot symptoms. Mol. Breed..

[40] Wan Q., Hongbo S., Zhaolong X., Jia L., Dayong Z., Yihong H. (2017). Salinity Tolerance Mechanism of Osmotin and Osmotin-like Proteins: A Promising Candidate for Enhancing Plant Salt Tolerance. Curr. Genom..

[41] Liu J., Han D., Shi Y. (2019). Gene Cloning, Expression, and Antifungal Activities of Permatin from Naked Oat (*Avena nuda*). Probiotics Antimicrob. Proteins.

[42] Skadsen R.W., Sathish P., Kaeppler H.F. (2000). Expression of thaumatin-like permatin PR-5 genes switches from the ovary wall to the aleurone in developing barley and oat seeds. Plant Sci..

[43] Thimme Gowda C., Purama S.N.S., Kammara R. (2020). TLPdb: A Resource for Thaumatin-Like Proteins. Protein J..

[44] Osmond R.I., Hrmova M., Fontaine F., Imberty A., Fincher G.B. (2001). Binding interactions between barley thaumatin-like proteins and (1,3)-beta-D-glucans. Kinetics, specificity, structural analysis and biological implications. Eur. J. Biochem..

[45] Snowdon A.L. (1990). A Colour Atlas of Post-Harvest Diseases and Disorders of Fruits and Vegetables. Volume 1: General Introduction and Fruits.

[46] Akhter A., Hage-Ahmed K., Soja G., Steinkellner S. (2016). Potential of Fusarium wilt-inducing chlamydospores, in vitro behaviour in root exudates and physiology of tomato in biochar and compost amended soil. Plant Soil.

[47] Gálvez L., Urbaniak M., Waśkiewicz A., Stępień Ł., Palmero D. (2017). *Fusarium proliferatum*—Causal agent of garlic bulb rot in Spain: Genetic variability and mycotoxin production. Food Microbiol..

[48] Chand S.K., Nanda S., Mishra R., Joshi R.K. (2017). Multiple garlic (*Allium sativum* L.) microRNAs regulate the immunity against the basal rot fungus *Fusarium oxysporum* f. sp. *cepae*. Plant Sci..

[49] Rout E., Nanda S., Joshi R.K. (2016). Molecular characterization and heterologous expression of a pathogen induced PR5 gene from garlic (*Allium sativum* L.) conferring enhanced resistance to necrotrophic fungi. Eur. J. Plant Pathol..

[50] Anisimova O.K., Shchennikova A.V., Kochieva E.Z., Filyushin M.A. (2021). Pathogenesis-Related Genes of PR1, PR2, PR4, and PR5 Families Are Involved in the Response to *Fusarium* Infection in Garlic (*Allium sativum* L.). Int. J. Mol. Sci..

[51] Smole U., Bublin M., Radauer C., Ebner C., Breiteneder H. (2008). Mal d 2, the thaumatin-like allergen from apple, is highly resistant to gastrointestinal digestion and thermal processing. Int. Arch. Allergy Immunol..

[52] Palacín A., Tordesillas L., Gamboa P., Sanchez-Monge R., Cuesta-Herranz J., Sanz M.L., Barber D., Salcedo G., Díaz-Perales A. (2010). Characterization of peach thaumatin-like proteins and their identification as major peach allergens. Clin. Exp. Allergy.

[53] Gavrović-Jankulović M., Spasić M., Cirković Velicković T., Stojanović M., Inić-Kanada A., Dimitrijević L., Lindner B., Petersen A., Becker W.M., Jankov R.M. (2008). Quantification of the thaumatin-like kiwi allergen by a monoclonal antibody-based ELISA. Mol. Nutr. Food Res..

[54] Nakashima K., Yamaguchi-Shinozaki K. (2013). ABA signaling in stress-response and seed development. Plant Cell Rep..

[55] Wang C., Lin T., Wang M., Qi X. (2020). An AC-Rich Bean Element Serves as an Ethylene-Responsive Element in Arabidopsis. Plants.

[56] Vicente-Carbajosa J., Moose S.P., Parsons R.L., Schmidt R.J. (1997). A maize zinc-finger protein binds the prolamin box in zein gene promoters and interacts with the basic leucine zipper transcriptional activator Opaque2. Proc. Natl. Acad. Sci. USA.

[57] Xu Z., Sun M., Jiang X., Sun H., Dang X., Cong H., Qiao F. (2018). Glycinebetaine Biosynthesis in Response to Osmotic Stress Depends on Jasmonate Signaling in Watermelon Suspension Cells. Front. Plant Sci..

[58] Geffers R., Sell S., Cerff R., Hehl R. (2001). The TATA box and a Myb binding site are essential for anaerobic expression of a maize GapC4 minimal promoter in tobacco. Biochim. Biophys. Acta.

[59] Zhao J.P., Jiang X.L., Zhang B.Y., Su X.H. (2012). Involvement of microRNA-mediated gene expression regulation in the pathological development of stem canker disease in *Populus trichocarpa*. PLoS ONE.

[60] Filyushin M.A., Anisimova O.K., Kochieva E.Z., Shchennikova A.V. (2021). Genome-Wide Identification and Expression of Chitinase Class I Genes in Garlic (*Allium sativum* L.) Cultivars Resistant and Susceptible to *Fusarium proliferatum*. Plants.

[61] Hauenschild F., Favre A., Schnitzler J., Michalak I., Freiberg M., Muellner-Riehl A.N. (2017). Spatio-temporal evolution of *Allium* L. in the Qinghai-Tibet-Plateau region: Immigration and in situ radiation. Plant Divers..

[62] Fritsch R.M., Friesen N., Rabinowitch H.D., Currah L. (2002). Evolution, domestication and taxonomy. Allium Crop Science: Recent Advances.

[63] Taylor A., Vagany V., Barbara D.J., Thomas B., Pink D.A.C., Jones J.E., Clarkson J.P. (2013). Identification of differential resistance to six *Fusarium oxysporum* f. sp. *cepae* isolates in commercial onion cultivars through the development of a rapid seedling assay. Plant Pathol..

[64] Borde M., Dudhane M., Jite P. (2012). Growth, water use efficiency and antioxidant defense responses of mycorrhizal and non mycorrhizal *Allium sativum* L. under drought stress condition. Ann. Plant Sci..

[65] Son J.H., Park K.C., Lee S.I., Kim H.H., Kim J.H., Kim S.H., Kim N.S. (2012). Isolation of cold-responsive genes from garlic, *Allium sativum*. Genes Genomes.

[66] Sun X., Zhu S., Li N., Cheng Y., Zhao J., Qiao X., Lu L., Liu S., Wang Y., Liu C. (2020). A Chromosome-Level Genome Assembly of Garlic (*Allium sativum*) Provides Insights into Genome Evolution and Allicin Biosynthesis. Mol. Plant.

[67] Anisimova O.K., Seredin T.M., Danilova O.A., Filyushin M. (2021). First Report of *Fusarium proliferatum* Causing Garlic clove Rot in Russian Federation. Plant Dis..

[68] Liu H., Lyu H.M., Zhu K., Van de Peer Y., Max Cheng Z.M. (2021). The emergence and evolution of intron-poor and intronless genes in intron-rich plant gene families. Plant J..

[69] Kim Y.S., Park J.Y., Kim K.S., Ko M.K., Cheong S.J., Oh B.J. (2002). A thaumatin-like gene in nonclimacteric pepper fruits used as molecular marker in probing disease resistance, ripening, and sugar accumulation. Plant Mol. Biol..

[70] Armentia A., Martín-Armentia S., Pineda F., Martín-Armentia B., Castro M., Fernández S., Moro A., Castillo M. (2020). Allergic hypersensitivity to garlic and onion in children and adults. Allergol. Immunopathol..

[71] Zipfel C., Felix G. (2005). Plants and animals: A different taste for microbes?. Curr. Opin. Plant Biol..

[72] Akbudak M.A., Yildiz S., Filiz E. (2020). Pathogenesis related protein-1 (PR-1) genes in tomato (*Solanum lycopersicum* L.): Bioinformatics analyses and expression profiles in response to drought stress. Genomics.

[73] Zhang Y., Chen W., Sang X., Wang T., Gong H., Zhao Y., Zhao P., Wang H. (2021). Genome-Wide Identification of the Thaumatin-like Protein Family Genes in Gossypium barbadense and Analysis of Their Responses to *Verticillium dahliae* Infection. Plants.

[74] Su L., Zhao X., Geng L., Fu L., Lu Y., Liu Q., Jiang X. (2021). Analysis of the thaumatin-like genes of *Rosa chinensis* and functional analysis of the role of *RcTLP6* in salt stress tolerance. Planta.

[75] Verma V., Ravindran P., Kumar P.P. (2016). Plant hormone-mediated regulation of stress responses. BMC Plant Biol..

[76] Fujii H., Zhu J.K. (2012). Osmotic stress signaling via protein kinases. Cell Mol. Life Sci..

[77] Deihimi T., Niazi A., Ebrahimi M., Kajbaf K., Fanaee S., Bakhtiarizadeh M.R., Ebrahimie E. (2012). Finding the undiscovered roles of genes: An approach using mutual ranking of coexpressed genes and promoter architecture-case study: Dual roles of thaumatin like proteins in biotic and abiotic stresses. Springerplus.

[78] Zhang X., Wang H., Zhu W., Li W., Wang F. (2020). Transcriptome Analysis Reveals the Effects of Chinese Chive (*Allium tuberosum* R.) Extract on *Fusarium oxysporum* f. sp. *radicis-lycopersici* Spore Germination. Cur. Microbiol..

[79] Zuo G.W., Li C.Y., Li B., Wei Y.R., Hu C.H., Yang Q.S., Yang J., Sheng O., Kuang R.B., Deng G.M. (2015). The toxic mechanism and bioactive components of Chinese leek root exudates acting against *Fusarium oxysporum* f. sp. *cubense*, tropical race 4. Eur. J. Plant Pathol..

[80] Abdelrahman M., El-Sayed M., Sato S., Hirakawa H., Ito S.I., Tanaka K., Mine Y., Sugiyama N., Suzuki Y., Yamauchi N. (2017). RNA-sequencing-based transcriptome and biochemical analyses of steroidal saponin pathway in a complete set of *Allium fistulosum*-*A. cepa* monosomic addition lines. PLoS ONE.

[81] Kumar S., Stecher G., Tamura K. (2016). MEGA7: Molecular evolutionary genetics analysis version 7.0. Mol. Biol. Evol..

[82] Hu B., Jin J., Guo A.Y., Zhang H., Luo J., Gao G. (2015). GSDS 2.0: An Upgraded Gene Feature Visualization Server. Bioinformatics.

[83] Babicki S., Arndt D., Marcu A., Liang Y., Grant J.R., Maciejewski A., Wishart D.S. (2016). Heatmapper: Web-enabled heat mapping for all. Nucleic Acids Res..

[84] Sugui J.A., Deising H.B. (2002). Isolation of infection-specific sequence tags expressed during early stages of maize anthracnose disease development. Mol. Plant Pathol..

